# Ag-Incorporated Polydopamine/Tannic Acid Coating on Titanium With Enhanced Cytocompatible and Antibacterial Properties

**DOI:** 10.3389/fbioe.2022.877738

**Published:** 2022-03-22

**Authors:** Hao Zhang, Xiaolong Shen, Zhikui Fei, Xingping Fan, Lan Ma, Haibo Wang, Congxue Tian, Bo Zhang, Rifang Luo, Yunbing Wang, Shengtian Huang

**Affiliations:** ^1^ School of Vanadium and Titanium, School of Biological and Chemical Engineering, Panzhihua University, Panzhihua, China; ^2^ National Engineering Research Center for Biomaterials, Sichuan University, Chengdu, China; ^3^ Material Corrosion and Protection Key Laboratory of Sichuan Province, Sichuan University of Science and Engineering, Zigong, China

**Keywords:** titanium, antibacterial, silver, tannic acid, cytotoxicity

## Abstract

Titanium (Ti) and its alloys are the most commonly used materials for bone implants. However, implant failure often happens due to bacterial infection. Developing antibacterial coatings on Ti implants is an effective strategy. Dopamine and tannic acid were cross-linked to form coating on Ti through Michael addition and Schiff base reaction. In addition, the Ag ions were grafted on the coating by the redox reaction of phenolic hydroxyl groups. Thus, an Ag-incorporated polydopamine/tannic acid coating was prepared on Ti substrate. SEM, EDS, water contact angle, FTIR, and XRD results demonstrated that the coating was formed on Ti successfully. The antibacterial activity of the coating against Gram-negative *E. coli* was examined, and the cytotoxicity of the coating was investigated by mouse fibroblast cells. The improvement of hydrophilicity, good cytocompatibility, and antibacterial effectiveness indicates that the coating has potential to surface modification of Ti implants.

## Introduction

Due to the favorable stability, mechanical properties, and biocompatibility, titanium and its alloys are the most commonly used materials for implants in dental and orthopedic (Elias et al., 2008; Suer et al., 2014; Scribante et al., 2018; Spriano et al., 2018). However, among titanium implant failures, bacterial infection and lack of osseointegration are the main causes (Jemat et al., 2015; Chouirfa et al., 2019). Titanium and its alloys have no antibacterial property and are sensitive to bacterial adhesion, which tends to bacterial infection (Spriano et al., 2018). Due to the presence of bacterial infection, a chronic inflammatory response may also develop at the infection site (Esposito and Leone, 2008; McConoughey et al., 2014; Høiby et al., 2015). In addition, titanium and its alloys are biologically inert, which made them that they can only be combined with the human body through physical chimerism. Such a combination lacks stability and may easily cause looseness and falloff in long-term use. Therefore, improving the antibacterial performance and osseointegration of Ti implants is necessary to improve the success rate of clinical implantation.

Surface modification is widely used to endow titanium and its alloys with osseointegration and antibacterial ability (Kulkarni et al., 2015; Ferraris and Spriano, 2016; Cheng et al., 2020; He et al., 2021). To achieve good osseointegration, the implants should trigger specific biological reactions at the interface to promote the combination between the tissue and the material (Hench et al., 1971). Ideal antibacterial coatings should have proven antibacterial effects with no toxicity as well as easy to manufacture (Orapiriyakul et al., 2018). Surface modification of physical adsorption and chemical covalent conjugation with antibacterial entities [such as antibiotics (Cabal et al., 2012; Lee et al., 2013; Park et al., 2014), antibacterial metal elements (Mei et al., 2014; Janković et al., 2015; He et al., 2021), and antimicrobial peptides (Bronk et al., 2014; Chen et al., 2014)] has become a practical method to prepare antibacterial coatings on Ti implant. However, researchers still show continued interest in fabricating universal antibacterial coatings through simple and low-cost methods. Compared with most research studies, the preparation of Ag-incorporated polydopamine/tannic acid coating requires only simple soaking, which is easy to operate and low cost. Mussel-inspired coating has great potential for universal surface modification. Dopamine can form coatings on various substrates through self-polymerization by a simple dip process. Moreover, studies have shown that tea polyphenols extracted from plants have anti-inflammatory, antibacterial, and antioxidant properties and can improve the mineralization and differentiation of osteoblasts (Chen and Dou, 2008; Bancirova, 2010; Ferruzzi, 2010; Carloni et al., 2013; Srivastava et al., 2013; Vester et al., 2014). Tea polyphenols contain a large number of catechol groups, which can react with dopamine by Michael addition and Schiff base reaction (Xu et al., 2016). Using this reaction, tea polyphenols can be introduced into the dopamine coating. Studies have reported that green tea polyphenols have a stimulating effect on bone-forming cells. Surface treatment of titanium alloy (Ti6Al4V) by tea polyphenols can induce hydroxyapatite deposition in simulated body fluid, showing that polyphenols can improve cell differentiation and stimulate biomineralization (Bancirova, 2010). However, the antibacterial ability of a single polyphenol coating is insufficient, and it is usually necessary to combine it with antibacterial agents. Among them, Ag has attracted much attention due to its broad spectrum of antibacterial ability. Although Ag has cytotoxicity, the balance between effective antibacterial activity and cytotoxicity can be achieved by controlling its loading dose in the coating. Cheng et al. prepared TiO_2_ nanotube (NT) by anodic oxidation on the Ti surface and then produced AgNT on the surface of NT through UV reduction of Ag ions. Ag-loaded TiO_2_-NT showed strong antibacterial activity against *Staphylococcus aureus* (MRSA, AT43300) *in vitro* and maintained for at least 30 days (Cheng et al., 2014). Bai et al. prepared Ti–Ag coating with AgNPs on Ti by pulsed DC magnetron sputtering, which could kill *Staphylococcus aureus* and was valid for more than 75 days (Bai et al., 2015). Zhang et al. used 50 nm AgNP in the dopamine-modified alginate/chitosan (DAL/CHI) polyelectrolyte multilayer for surface modification of titanium alloys. The DAL/CHI layer can improve the wetting ability of titanium alloy and significantly promote the proliferation of fibroblasts. After incorporation of AgNP, although the L929 cell activity slightly decreased, the growth of *Escherichia coli* and *Staphylococcus aureus* was significantly inhibited (Zhang et al., 2013).

Most polyphenols can be complexed with metal ions and form stable complexes, such as Ag (I) and Cu (II) (Leopoldini et al., 2011; Rodríguez et al., 2016). Ag ions can be grafted onto the surface of medical titanium alloys by polyphenol coating due to their ability to complex metal ions. The electron-donating ortho-phenolic hydroxyls of polyphenol can serve as a reducing agent to reduce Ag^+^ to Ag^0^ (Huang et al., 2016). Tannic acid (TA) is a natural reducing agent, which is widely present in plants, has health benefits such as chemoprevention and antioxidant activity. The rich catechol groups of TA could reduce metal ions to metal nanoparticles in the solution (Sagbas et al., 2015; Luo et al., 2016). Furthermore, the catechol groups of TA could cross-link with amino groups of dopamine to form coatings on various substrates by Michael addition and Schiff base reaction (Luo et al., 2013; Zhang et al., 2018).

In this study, the Ag-incorporated polydopamine/tannic acid coating on the surface of Ti substrate was constructed to endow Ti implants with antibacterial properties. First, dopamine and tannic acid are co-deposited to construct a phenol–amine cross-linked coating on the titanium surface. During the co-deposition process, the phenolic hydroxyl groups of tannic acid were oxidized into quinones. The quinones can react with amine groups in dopamine via Michael addition and Schiff base reaction to construct covalent bonding of dopamine and tannic acid. Then, the Ag ions were grafted on the coating by the redox reaction of phenolic hydroxyl groups. The complexed Ag on the coating can effectively improve the antibacterial ability and avoid problems such as delayed inflammation. The surface morphology, hydrophilicity, surface chemical properties, antibacterial activity, and cytocompatibility of the coating were investigated.

## Materials and Methods

### Preparation of Samples

**SCHEME 1:**
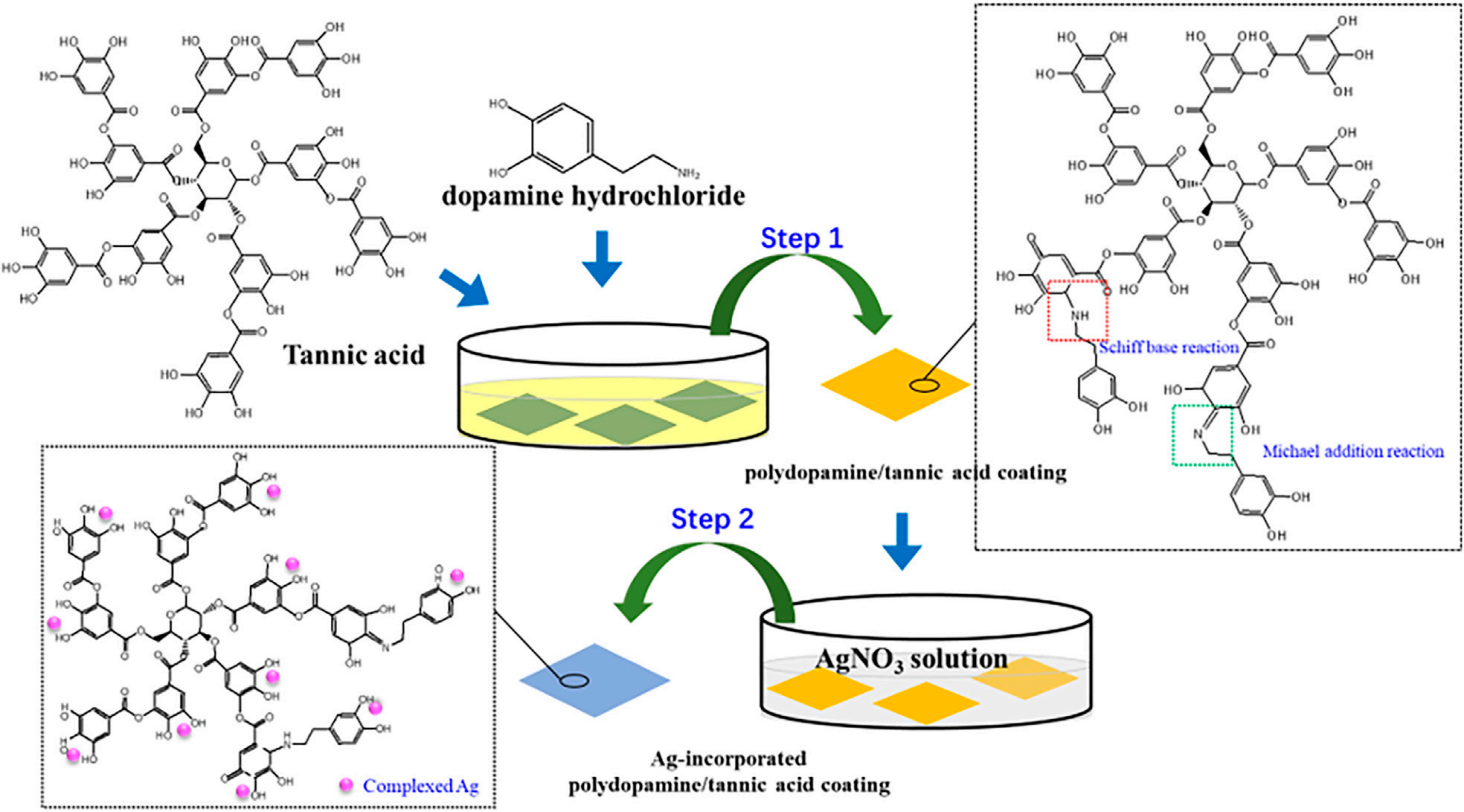
Experimental procedure and possible reaction mechanism of Ag-incorporated polydopamine/tannic acid coating.

Pure titanium plates (99.6 at%, Grade 2) were cut into rectangular samples with dimensions of 10 mm × 10 mm × 1 mm. The samples were polished to 2000 # grit level, ultrasonically cleaned with acetone, alcohol, and ultrapure water for 15 min each, and then dried at 40°C. To prepare polydopamine/tannic acid-decorated titanium (Ti-PT), 30 mg of tannic acid (Wuxi Taiyo Green Power Co., Ltd., Jiangsu, China) and 30 mg dopamine hydrochloride (Sigma-Aldrich Chemical Co.) were dissolved in 30 ml Tris–HCl solution (10 mM, pH = 8.5), and then the Ti substrates were immersed in the solution at 20°C for 24 h. Then, the samples were ultrasonically cleaned in ultrapure water and dried at 40°C. In order to further load Ag ions on the Ti-PT, the PT-coated samples were avoid-light placed in silver nitrate solution (AgNO_3_, 0.1 mg/ml) at 20°C for 1 day. The obtained sample was denoted as Ti-PT-0.1Ag. Ag is a commonly used antibacterial metal, but high concentrations can cause cytotoxicity. In order to obtain the optimal process, three different AgNO_3_ solution concentrations were set up to compare antibacterial activity and cytotoxicity. The Ti-PT samples were also immersed in 0.25 mg/ml and 0.5 mg/ml AgNO_3_ solution, and the prepared samples were named Ti-PT-0.25Ag and Ti-PT-0.5Ag, respectively. The experimental procedure including the preparation of the polydopamine/tannic acid coating, complexation of Ag^+^, and possible reaction mechanism are schematically illustrated in Scheme 1.

### Surface Characterization

FESEM (field emission scanning electron microscopy, JSM-7401F, JEOL, Japan) was used to observe the micromorphologies of samples. EDS (energy-dispersive X-ray spectrometry) was used to provide the element composition of the coatings. The optical contact angle system (Model SL200A/B/D) was employed to test the water contact angles of the samples by using a 2-μl deionized water droplet at room temperature. The attenuated total reflection FTIR infrared spectroscopy with a scan scope from 4,000 to 500 cm^−1^ was employed for chemical characterization analysis of the coatings. The changes in the chemical states and the high resolution spectra of C 1s, O 1s, N 1s and Ag 3d were analyzed with an X-ray photoelectron spectroscopy system (XPS, XSAM800, Kratos Ltd., United Kingdom).

### Antibacterial Activity

The antibacterial capability of the samples was assessed by *Escherichia coli* (*E. coli*; ATCC 25922). The agar plating of colonies was carried out to investigate the bactericidal ability. Ti, Ti-PT, Ti-PT-0.1Ag, Ti-PT-0.25Ag, and Ti-PT-0.5Ag were sterilized by ultraviolet for 24 h. Each sample was incubated with 400 μl of solution containing *E. coli* suspension (1 × 10^6^ CFU ml^−1^) at 37°C for 24 h. Then, the bacterial suspension was collected into the sterilized centrifugal tube with 4 ml physiological saline. The bacteria in the tube from different samples (Ti, Ti-PT, and Ti-PT-Ag) were dissociated using a vortex mixer. The dissociated bacteria solution was diluted with physiological saline and then added into the standard Luria–Bertani agars and incubated at 37°C for 24 h. The bacteria concentration of *E. coli* re-cultured on agars after dissociation from the samples was detected.

### Cytotoxicity Test

The mouse fibroblast cell line L929 was chosen for measuring the cytotoxicity of PT-Ag coating. Ti, Ti-PT, Ti-PT-0.1Ag, Ti-PT-0.25Ag, and Ti-PT-0.5Ag were sterilized by exposure to UV light overnight and then placed in 24-well plates. The fibroblast suspension (density 2 × 10^4^ cells/cm^2^) was seeded onto the samples and incubated for 1 day in 5% CO_2_ at 37°C; the plates without samples were used as a blank control. CCK-8 assay was used to determine the cell viability. Subsequently, the samples were washed by phosphate buffer saline (PBS) three times and stained by rhodamine 123. The cells on the samples were visualized using an inverted fluorescence microscope (Leica, Germany).

## Results and Discussion

### Surface Characterization

**FIGURE 1:**
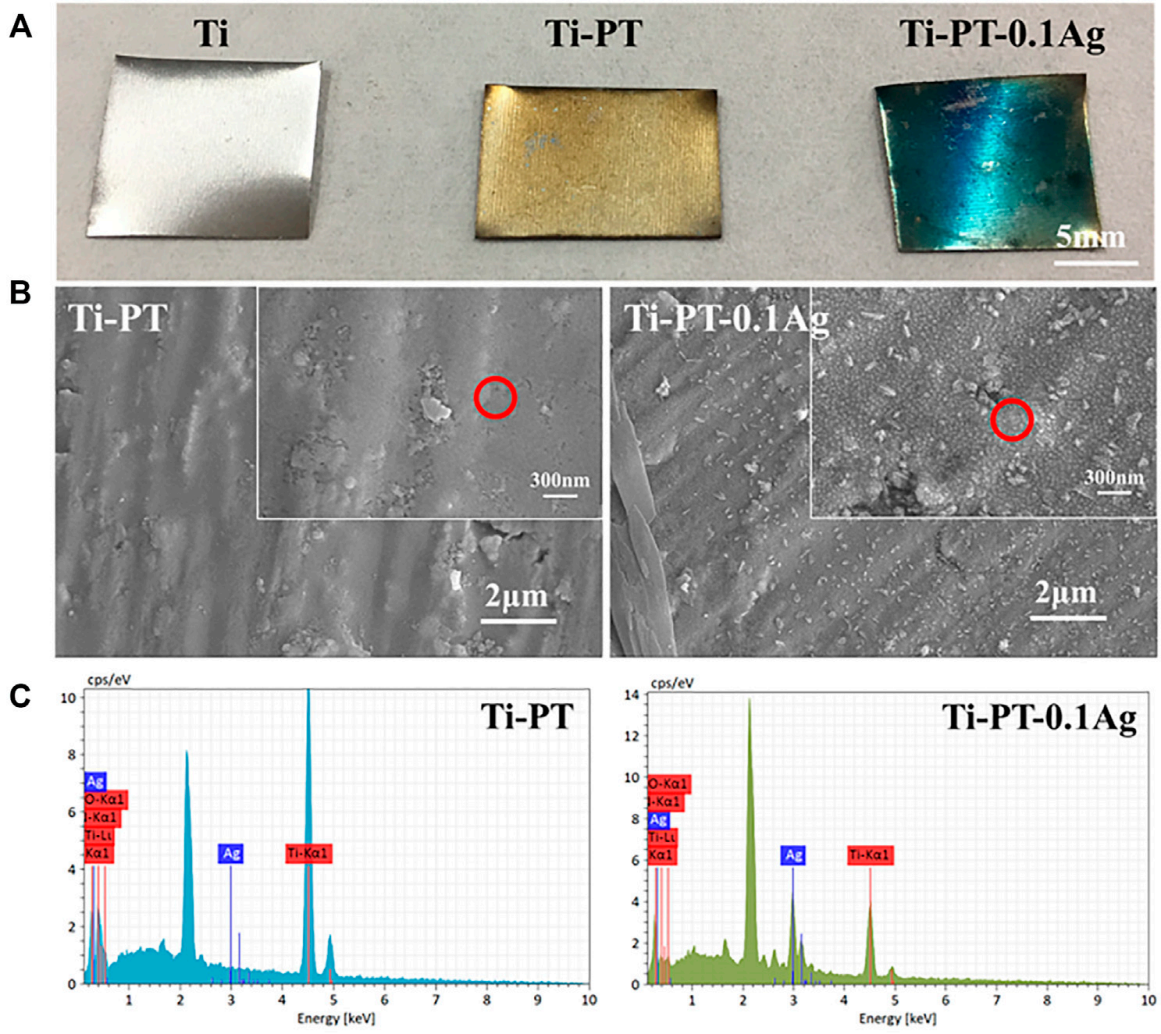
**(A)** Digital pictures of Ti, Ti-PT, and Ti-PT-0.1Ag samples. **(B)** Surface morphology and microstructure of Ti-PT and Ti-PT-0.1Ag. **(C)** EDS spectra from the position marked by red circle.

The optical images, SEM images, and the surface elemental composition of samples were studied and shown in Figure 1. As shown in Figure 1A, compared with bare Ti, the PDA/TA-coated surface (Ti-PT), especially the coatings grafted with Ag^+^ (Ti-PT-0.1Ag) have obvious color change. The Ti-PT appears brown due to the presence of catechol and amine groups. It is speculated that after the phenol oxidized to quinone, the Michael addition reaction and Schiff base reaction occur with the amine donor, and the polymerization and cross-linking process is manifested by color. After immersing in silver nitrate solution, the surface of Ti-PT-0.1Ag turned blue due to the complexation of phenolic hydroxyl and silver ions. The color change of the sample surface preliminary indicates that the coating can be deposited on the Ti surface.

Figure 1B indicates SEM images of the Ti-PT and Ti-PT-0.1Ag. It can be clearly seen that the Ti-PT exhibited film-covered morphology. The addition of Ag^+^ ion to the PT coating caused some particle depositions, and the size of the particles is about 30 nm. The catechol and hydroxyl groups on Ti-PT have the ability to complex and reduce Ag^+^ ions to form metal silver. The elemental compositions of the surface were detected by EDS analysis (Figure 1C). The C, O, and Ti elements were detected on the Ti-PT and Ti-PT-0.1Ag, while the Ag element appeared on the surface of Ti-PT-0.1Ag. The EDS analysis confirmed the precipitated particles on Ti-PT-0.1Ag as silver. The presence of silver on Ti-PT-0.1Ag indicates the successful loading of silver on the coating.

**FIGURE 2:**
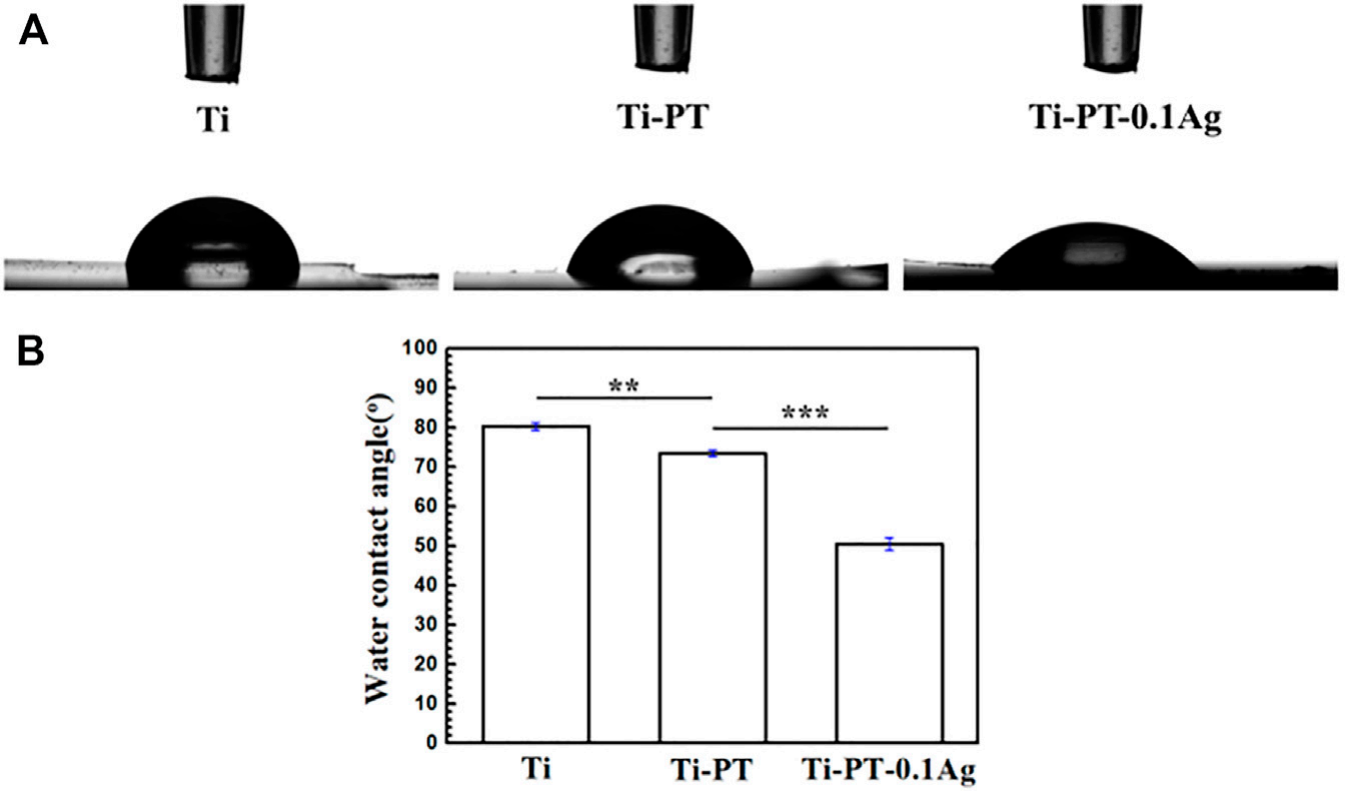
**(A)** Shapes of water droplets on the Ti, Ti-PT, and Ti-PT-0.1Ag. **(B)** Static water contact angles of Ti, Ti-PT, and Ti-PT-0.1Ag. Data are presented as mean ± SD and analyzed by one-way ANOVA (**p < 0.01, ***p < 0.001).

The relatively hydrophilic surface has higher surface energy, which promotes the adhesion of cells, thus increasing the osteointegration ability between the implant and the host bone tissue. The hydrophilicity of the surface was characterized by the water contact angle, shown in Figure 2. The contact angle decreased from 80.13° ± 0.64° on the Ti surface to 72.50° ± 0.87° on the Ti-PT surface. The incorporated PDA/TA coating altered the contact angle on the Ti obviously, owing to the phenol and amino groups on the surface. After being grafted with Ag^+^ ions, the contact angle of Ti-PT-0.1Ag further decreased to 50.14° ± 2.72°. These results indicate that the hydrophilicity is improved after surface modification.

**FIGURE 3:**
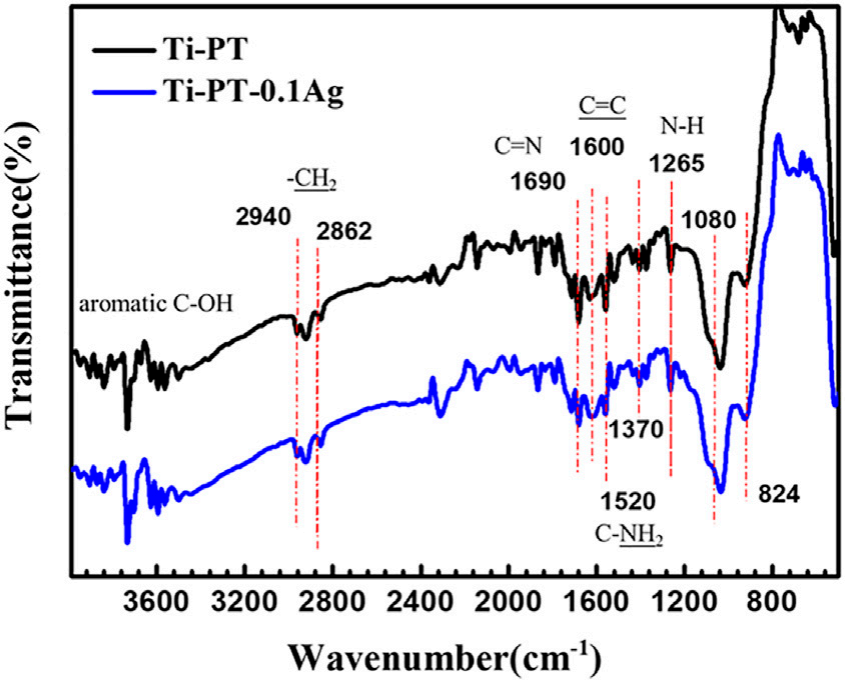
FTIR spectra of Ti-PT and Ti-PT-0.1Ag.

**FIGURE 4:**
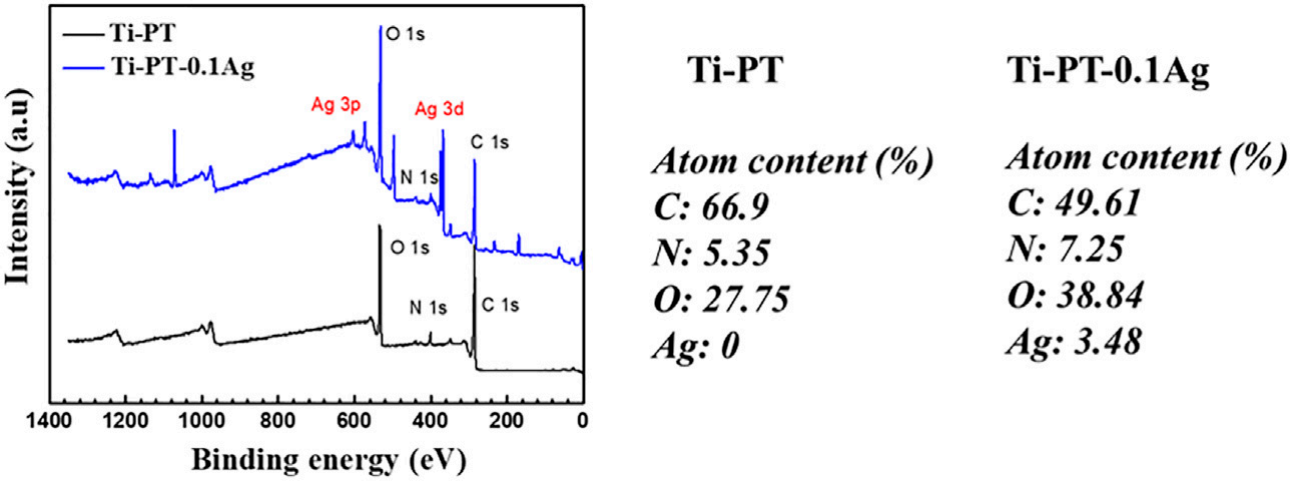
XPS spectra survey for Ti-PT and Ti-PT-0.1Ag surfaces with the surface elemental composition.

In order to confirm the cross-linking of dopamine and tannic acid and identify the grafting principle of silver, FTIR infrared spectroscopy (Figure 3) and XPS (Figure 4) were used for further analysis. FTIR spectra exhibited the characteristic bands of Ti-PT and Ti-PT-0.1Ag, as shown in Figure 3. The basic feature peaks of dopamine and tannic acid were well preserved on both the surface of Ti-PT and Ti-PT-0.1Ag. The peaks around 3,500 cm^−1^ are the phenolic hydroxyl groups. The peaks at 2,940 and 2,862 cm^−2^ are C-H stretching vibration (Chen et al., 2015). The peak at 1,600 cm^−1^ represents the aromatic nucleus, derived from the benzene ring structure of the dopamine and tannic acid. The formation of the vibration peak of aromatic O-H (1,265 cm^−1^) and the stretching vibration peak of C-O (1,370 cm^−1^) from the phenolic group could be observed. In addition, the stretching vibration peak of aliphatic primary amine C-N (1,080 cm^−1^) and the N-H deformation vibration peak of primary amine (827 cm^−1^) from dopamine are shown (Zhang et al., 2019). It is noteworthy that the peaks at 1,690 cm^−1^ and 1,520 cm^−1^, which present C=N stretching vibration and N-H scissoring vibrations, respectively, confirmed the cross-link of dopamine and tannic acid. According to the analysis, although there is no significant difference in FTIR infrared spectroscopy between Ti-PT and Ti-PT-0.1Ag, detailed analysis is needed through XPS.

**FIGURE 5:**
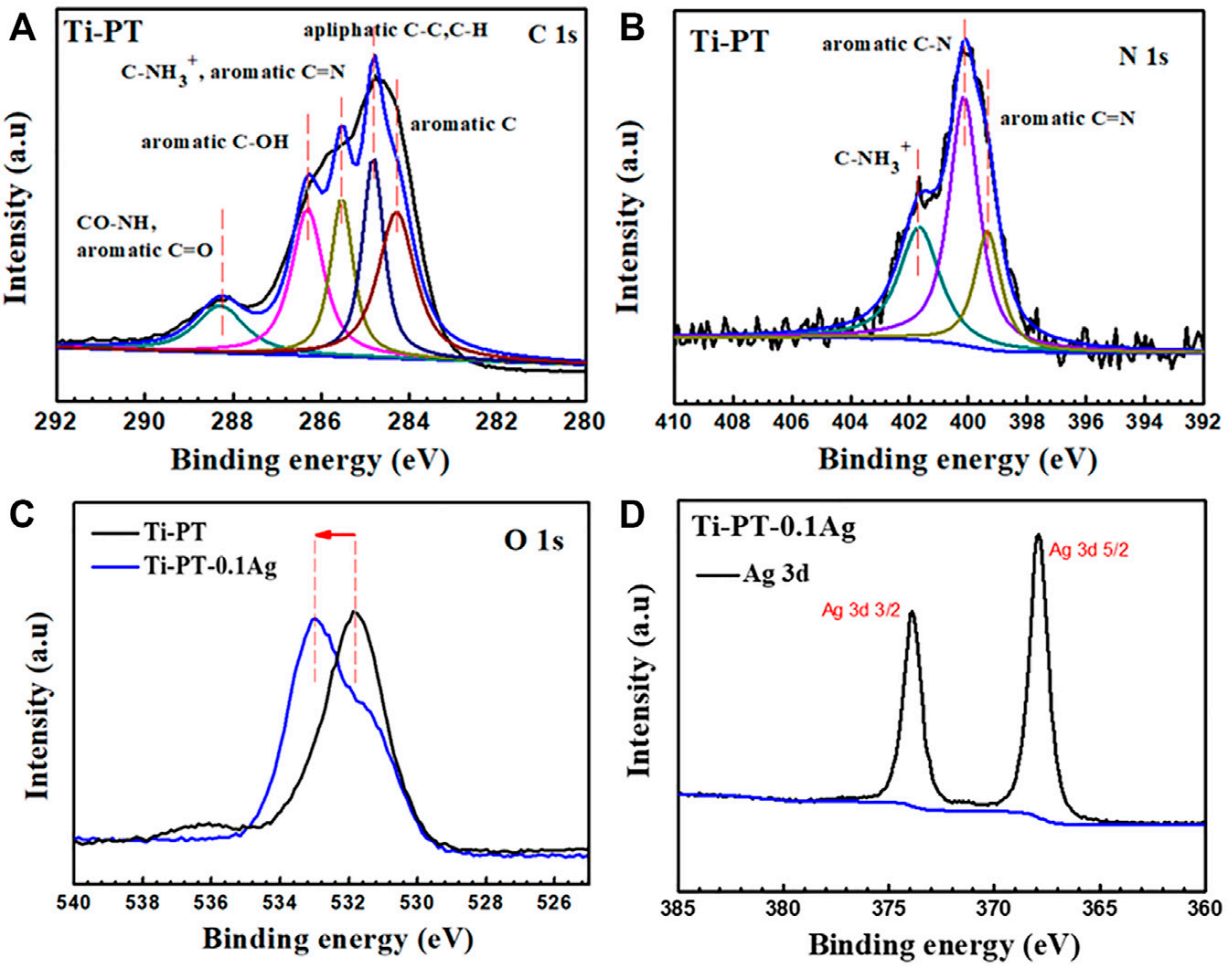
High-resolution XPS spectra and fitting results of C 1s **(A)**, N 1s **(B)**, O 1s **(C)**, and Ag 3d **(D)** on Ti-PT and Ti-PT-0.1Ag.

The XPS spectra survey of the surface was examined to further identify the presence of Ag ions and detect the chemical bonding states of the element on the surface. As shown in Figure 4, the Ti-PT and Ti-PT-0.1Ag showed the appearance of C 1s, O 1s, and N 1s. The signal peaks of ∼600 and ∼370 eV appeared on the Ti-PT-Ag, indicating successful grafting of Ag^+^ ions on the surface. The atom content ratios of C, N, O, and Ag on the surface of Ti-PT and Ti-PT-0.1Ag are also listed in Figure 4. The high-resolution spectra and fitting results of C 1s, N 1s, O 1s, and Ag 3d on samples are shown in Figure 5. The grafted Ag has little effect on the chemical bonding states of C and N elements; therefore, only the high-resolution spectra of C1s and N1s of the Ti-PT are analyzed, as shown in Figure 5A and Figure 5B. The C 1s exhibited five bonding states: 1) 288.3 eV corresponding to the aromatic C=O; 2) 286.4 eV corresponding to the aromatic C-OH; 3) 285.6 eV corresponding to C-NH_3_^+^, aliphatic C-N, and aromatic C-N; 4) 284.9 eV corresponding to aliphatic C-C and C-H; and 5) 284.2 eV corresponding to aromatic C (Cheng et al., 2014). It should be noted that the fitting result of C 1s is not unique, and uncertainty cannot be completely avoided during the fitting process. However, if the fitting results of C 1s, N 1s, and O 1s show consistency, it can be mutually corroborated. Figure 5B shows the high-resolution fitting result of N 1s, which exhibit three bonding states: C-NH_3_^+^ at 401.4 eV, aromatic C-N at 400.5 eV, and aromatic C=N at 399.6 eV. The aromatic C-N obtained by the Michael addition reaction and the aromatic C=N obtained by the Schiff base reaction proved that the dopamine and tannic acid had cross-linked in the coating. The high-resolution spectra of O 1s on Ti-PT and Ti-PT-0.1Ag are compared in Figure 5C. The peak position of O 1s on Ti-PT-0.1Ag shifted to a higher binding energy than that on Ti-PT. This is caused by the complexation of the phenolic hydroxyl group or quinone group with the silver ion, which proves the occurrence of the complexation reaction. The binding energy of Ag at ∼370 eV was amplified, and the peaks at 368.7 eV (3d 5/2) and 374.5 eV (3d 3/2) were found on Ti-PT-Ag, as shown in Figure 5D. The binding energy difference of nearly 6.0 eV between Ti-PT and Ti-PT-0.1Ag typically indicated the existence of Ag nanoparticles (Srivastava et al., 2013).

### Antibacterial Ability

**FIGURE 6:**
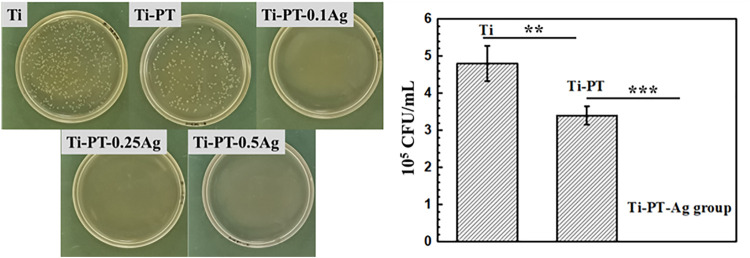
Photographs (left) and the number (right) of *E. coli* colonies formation on Ti, Ti-PT, Ti-P-0.1Ag, Ti-PT-0.25Ag, and Ti-PT-0.5Ag. Data are presented as mean ± SD and analyzed by one-way ANOVA (**p < 0.01, ***p < 0.001).

The detached bacteria from the samples cultivated for 24 h were re-cultured on agar plates. The bacterial colony formation and the number of *E. coli* colonies with Ti, Ti-PT, Ti-PT-0.1Ag, Ti-PT-0.25Ag, and Ti-PT-0.5Ag are shown in Figure 6. The agar plates of the bare Ti substrate displayed numerous bacterial colonies already after 24 h. Compared to those of Ti, significantly reduced bacteria were observed on those of the Ti-PT and Ti-PT-Ag groups. The tannic acid contained in the PT coating showed some antibacterial properties, especially the Ti-PT-Ag group, which showed 100% bacterial killing efficiency for *E. coli*. The silver normally exhibits antimicrobial properties, and the Ti-PT-Ag group showed antibacterial ability for *E. coli* within 12 h culture. The Ti-PT-0.1Ag group, with the lowest AgNO_3_ concentration of 0.1 mg/ml, has shown the ability to inhibit *E. coli* colonies effectively.

### Cytotoxicity

**FIGURE 7:**
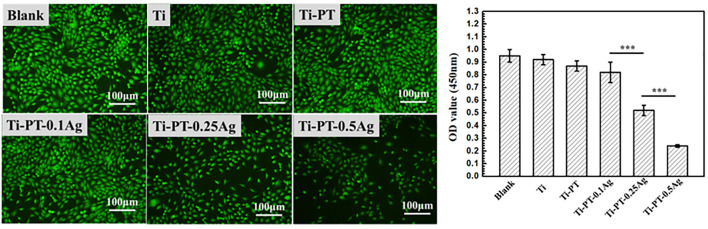
Morphology of mouse fibroblast cells adhered on various samples and the viability of cells attached onto the samples after culture for 1 day (×100 magnification). Data are presented as mean ± SD and analyzed by one-way ANOVA (***p < 0.001).

The biocompatibility of the implant is crucial. Silver grafted onto the coating has antibacterial effect, but excessive concentration can lead to cytotoxicity. Therefore, the mouse fibroblast cell was used for cytotoxicity evaluation of the samples. The typical fluorescence images and CCK value of cells cultured on samples for 1 day are shown in Figure 7. After 24 h culture, the viability of cell growth on Ti, Ti-PT, and Ti-PT-0.1Ag showed no significant difference, which is similar to that of blank. There was a decrease in cell number observed when cultured on the 0.1 mM-AgNO_3_-treated Ti metal surface. However, when fibroblast cells were cultured on the surface of Ti-PT-0.25Ag and Ti-PT-0.5Ag, the number and activity of cells were significantly reduced. The Ti-PT-0.1Ag did not cause significant cytotoxicity to fibroblast cells demonstrating its good biocompatibility. The results showed that the graft concentration of 0.1 mg/ml silver nitrate was safe, which not only had no toxic effect on human cells but also had effective antibacterial activity.

## Conclusion

In this work, an Ag-incorporated polydopamine/tannic acid coating was prepared on pure titanium via the simple immersion method. Initially, dopamine and tannic acid formed a self-polymerizing coating on pure titanium via Michael-type addition and Schiff base reaction. Then, the phenolic hydroxyl functional groups on the coating were used to complex Ag ions. The results demonstrated that Ti-PT-Ag possesses antibacterial ability and cytocompatibility compared with bare Ti and Ti-PT. The Ti-PT-0.1Ag showed no significant cytotoxicity toward the mouse fibroblast cell and improved antibacterial properties toward *E. coli*. The result showed that the balance between effective antibacterial activity and cytotoxicity can be achieved by controlling the Ag-loading dose in the coating. The Ag-incorporated polydopamine/tannic acid coating might be used as an antibacterial and biocompatible platform for potential orthopedic implants.

## Data Availability

The original contributions presented in the study are included in the article/Supplementary Material, further inquiries can be directed to the corresponding authors.
